# Immunomagnetic separation of *Toxoplasma gondii* and *Hammondia* spp. tissue cysts generated in cell culture

**DOI:** 10.3389/fvets.2022.1033380

**Published:** 2022-10-13

**Authors:** Mariana M. Rezende-Gondim, Aristeu V. da Silva, Jitender P. Dubey, Gereon R. M. Schares, Luís F. P. Gondim

**Affiliations:** ^1^Departamento de Anatomia, Patologia e Clínicas, Escola de Medicina Veterinária e Zootecnia, Universidade Federal da Bahia, Salvador, Bahia, Brazil; ^2^Departamento de Biologia, Universidade Estadual de Feira de Santana, Feira de Santana, Bahia, Brazil; ^3^United States Department of Agriculture, Agricultural Research Service, Beltsville Agricultural Research Center, Animal Parasitic Diseases Laboratory, Beltsville, MD, United States; ^4^Friedrich-Loeffler-Institut, Federal Research Institute for Animal Health, Institute of Epidemiology, National Reference Centre for Toxoplasmosis, Greifswald-Insel Riems, Germany

**Keywords:** monoclonal antibody, tissue cyst wall, *Toxoplasma gondii*, *Hammondia hammondi*, *Hammondia heydorni*, immunomagnetic

## Abstract

*Toxoplasma gondii* is commonly transmitted among animals and humans by ingestion of infected animal tissues or by consumption of food and water contaminated with environmentally-resistant oocysts excreted by cats. Tissue cysts and oocysts have different walls, whose structures and compositions are poorly known. Herein, we describe an immunomagnetic separation (IMS) method that was successfully used for purification of *T. gondii* tissue cysts generated in cell culture. We used an IgG monoclonal antibody (mAb) that reacts against antigens in tissue cyst walls. Many *in vitro* produced cysts were obtained by this IMS; >2,000 *T. gondii* cysts were isolated from a single culture flask of 25 cm^2^. Tissue cysts from two *Hammondia* spp., *H. hammondi*, and *H. heydorni*, produced in cell culture were also separated using this method. As a reference, purification of tissue cysts by Percoll gradients was used. Percoll was able to separate *T. gondii* tissue cysts produced in mice but was not suitable for purifying *T. gondii* tissue cysts produced *in vitro*. The IMS described here should favor proteomic studies involving tissue cysts of *T. gondii*.

## Introduction

*Toxoplasma gondii* is a globally distributed protozoan parasite, which can infect almost all warm-blooded animals, including humans (1). The two parasite stages involved in its oral horizontal transmission are tissue cysts (TC) and oocysts. Tissue cysts are formed in brain, muscles and other organs of mammalian and avian hosts; they may contain thousands of bradyzoites (2). Animals and humans are mainly infected by consuming TC in raw or undercooked animal tissues and oocysts in contaminated food or water. Other ways of transmission include transplacental infection, organ transplantation, blood transfusion, and accidental inoculation using needles, but the infection by ingestion of oocysts and TC are believed to occur more often (3).

Currently, there is no effective way to eliminate TC in live animals. Tissue cysts possess walls, whose composition and structures are poorly known. The TC wall is formed by a combination of molecules from the host cell and by proteins secreted by the parasite, that confers both resistance to the TC, as well as helps the parasite to evade the host immune system (4, 5). Several proteins have been identified in the TC wall, including a 65KDa protein abundant in its matrix (6), the CST1 protein, which is associated to the integrity of *in vivo* produced TC (7), and BCP1, which is also essential to cyst wall formation (8). A study using a promiscuous biotin ligase allowed the identification of previously described cyst wall proteins of *T. gondii*, as well as undescribed ones (9).

A crucial step to better understand the composition of the TC wall of *T. gondii* is to obtain purified TC. A monoclonal antibody (mAb) initially established to bind oocysts and designated K8/15-15 was shown to also bind to TC walls of *T. gondii* (10). In addition, this mAb also binds to cyst walls of related coccidia, including *Neospora caninum, Hammondia hammondi*, and *Hammondia heydorni* (10). In the present study, we describe an immunomagnetic separation (IMS) method to obtain purified TC of *T. gondii*. In addition, the IMS was also qualitatively tested to capture *in vitro* produced TC of *H. hammondi* and *H. heydorni*.

## Materials and methods

### Study design

**Figure 1:**
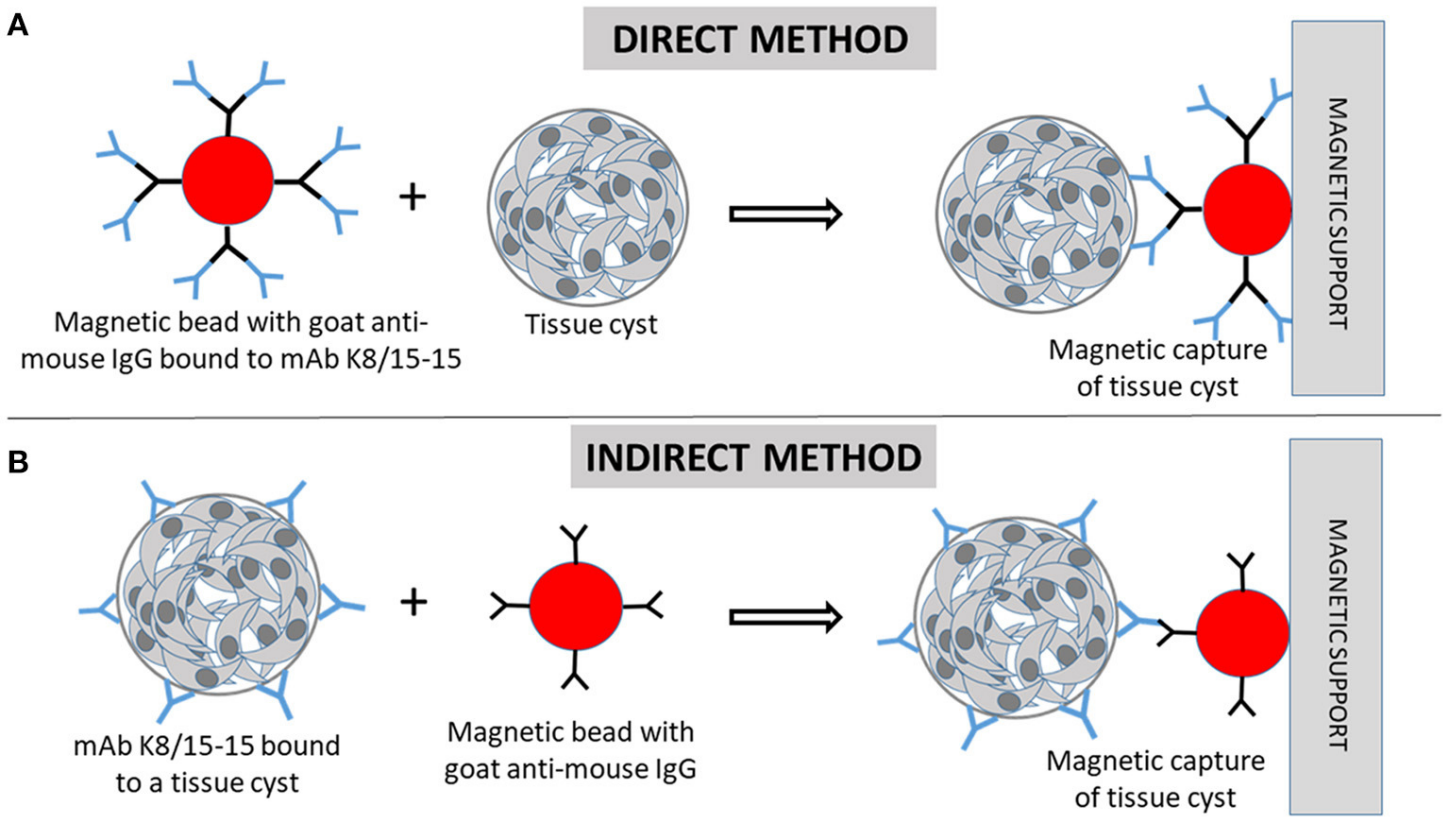
Immunomagnetic separation of *Toxoplasma gondii* tissue cysts (TC). **(A)** In the direct capture, magnetic beads coated with goat anti-mouse IgG are firstly incubated with the monoclonal antibody (mAb) K8/15-15. Then, the cell suspension containing TC are incubated with the beads. **(B)** In the indirect method, the cell suspension containing tissue cysts are firstly incubated with the mAb, followed by incubation with the magnetic beads. **(A,B)** The beads bound to TC are magnetically captured by the magnetic support (magnetic cell separator).

An IMS method was developed focusing the purification of *T. gondii* cysts produced in cell culture. The method was initially tested using *T. gondii* tissue cysts produced in mice, as well as *in vitro* generated cysts of *Hammondia* spp., which became available from a previous experiment (10). Subsequently, the IMS method was tested in different conditions (direct and indirect capture at two different temperatures) using *T. gondii* cysts generated in cell culture (Figure 1). Parasites were grown as tachyzoites in Monkey Kidney cells (Marc-145) and submitted to stress conditions to induce cyst formation. *In vitro* generated TC were tested by IMS and the captured cysts were quantified. Non-specific binding of host cells was tested with the magnetic beads and mAb. Purification of *in vitro* produced cysts was also attempted by Percoll gradients.

### Cell culture and *in vitro* production of cysts

Monkey kidney cells (Marc-145) (ATCC^®^ CRL-12231) were selected for this study, as these cells have been shown to resist to stress conditions during cell culture for *in vitro* production of *N. caninum* cysts (11). Marc-145 cells were cultured in RPMI medium supplemented with 1% antibiotic/antimycotic solution (100 units/mL of penicillin, 100 μg/mL of streptomycin and 0.25 μg/mL of amphotericin B) (Gibco^®^, Carlsbad, USA) and 5% of inactivate bovine serum (Invitrogen/Gibco^®^, Auckland, NZ), at 37°C in a humidified incubator containing 5% CO_2_.

For *T. gondii in vitro* cyst production, 7 × 10^5^ Marc-145 cells were placed in 25 cm^2^ flasks and after 48 h, 7 × 10^6^ tachyzoites of a chicken isolate of the parasite (TgCkBr284) (12, 13) were added to the flask; 24 h after infection, cultures were transferred to an incubator with no CO_2_ supply. Culture medium was replaced by alkalinized medium (pH 8.1) by adding 1M NaOH (18). Alkalinized medium was replaced every 24 h for four consecutive days. Cell monolayers were trypsinized, and after detachment of the cells from the flask surface, trypsin was blocked by adding 2 ml of fresh medium (RPMI with 1% antibiotic/antimycotic and 5% of inactivate bovine serum). The content from each flask was transferred to a 15 ml-tube. Aliquots were collected from each tube and placed on teflon-coated slides for immunofluorescence examination. These *in vitro* produced cyst suspensions were named “pre-capture” samples. The 15 ml-tubes were centrifuged (300 *g*, at 24°C, no brake), the supernatant discarded and the sediment used for IMS.

### Magnetic beads and magnetic particle separator

Magnetic beads with diameters of 4.5 μm and coated with goat anti-mouse IgG (Dynabeads^®^, Invitrogen by Life Technologies) were used. The antibodies attached to these beads can react with the heavy chain of mouse IgG. The minimal volume of beads per reaction suggested by the manufacturer is 25 μl (~1 × 10^7^ beads). However, we used 5 μl (~2 × 10^6^ beads) or 10 μl (~4 × 10^6^ beads) of the original bead suspension per reaction. In case of the production of 2,500 cysts in a culture flask, using 5 μl of beads would result in 800 of beads for each cyst of *T. gondii*. A magnetic particle separator (MPS) (MPG^®^ 3-in-1 MPS^®^, Lincoln Park, USA) which contains inserts for tubes of 1.5, 15, and 50 ml was used to bind beads.

### Washing and coupling magnetic beads to the monoclonal antibody K8/15-15

The method used to couple the mAb to anti-mouse IgG magnetic beads was executed similarly as suggested by the manufacturer but was slightly modified and adapted for 1.5 ml tubes. The original 5-ml vial containing the beads (4 × 10^8^ beads/ml) was vortexed for 40 s, and 25 μl (~1 × 10^7^ beads) were transferred to a 1.5-ml centrifuge tube. The beads were suspended in 1 ml of an isolation buffer (PBS free of Ca^2+^ and Mg^2+^ with 0.1% BSA and 2 mM EDTA, pH 7.4) and placed on a magnetic particle separator (MPS) for 1 min. The supernatant was discarded using an aspiration pump while the tube was still on the MPS. The tube was removed from the MPS, and the beads resuspended with 25 μl of isolation buffer.

Two hundred μl of the mAb K8/15-15 (hybridoma supernatant) at 1:10 or 1:5 dilutions in isolation buffer was homogenized with the beads (25 μl) and incubated at 7°C in a mixer with gentle titling of the tubes every 3–4 min. The tube was placed on the MPS for 1 min and the supernatant aspirated and discarded while the tube was on the MPS. The tube was removed from the MPS, and 1.5 ml of isolation buffer was added to wash the excess of unbound antibodies. The supernatant was removed while the tube was attached to the MPS by using a suction device. The washing step was repeated once. The tube was removed from the MPS, and the beads suspended with 1 ml of isolation buffer. This suspension containing the beads coupled to the mAb was stored at 4°C. At the end of the mAb-beads coupling procedure, the volume of beads in 1 ml was 6.4 × 10^6^, which was divided in fractions of 200 μl (1.26 × 10^6^ beads per aliquot) for subsequent experiments.

### IMS of tissue cysts produced in mice and cysts of related coccidia

Two C57 mice were each intraperitoneally inoculated with two tissue cysts of the ME-49 strain of *T. gondii*. Four months later, the mice were euthanized, and their brains aseptically removed. The two mice were used in a previous experiment (10), approved by the Landesamt für Landwirtschaft, Lebensmittelsicherheit und Fischerei of the German Federal State of Mecklenburg-Vorpommern. Each mouse brain was homogenized in 600–800 μl of PBS/T (0.05% Tween 20) using a glass tissue grinder and TC quantified by microscopically counting three aliquots of 10 μl of the brain suspension on a glass slide with a coverslip at 200 × magnification. The TC were concentrated by Percoll gradients in 15-ml plastic centrifuge tubes, as previously described (14, 15). In brief, 10 ml of PBS/T (0.05% Tween 20) were added to 1 ml of the brain suspension. Then, 1.5 ml of 30% Percoll in PBS/T and 1.5 ml of 90% Percoll in PBS/T were consecutively underlayered to the bottom of the brain suspension. The tube was centrifuged at 1,500 *g* at 4°C for 15 min. The entire content of the 30 and 90% Percoll gradients were collected, added to a 50 ml centrifuge tube, and the tube filled with PBS to the top. The tube was centrifuged for 1,200 *g* for 10 min at 4°C, the supernatant discarded, and the sediment resuspended with 200 μl of PBS.

The TC, which had been purified by Percoll gradients, were mixed with 200 μl of isolation buffer. Each fraction of tissue cysts was added to a 1.5-ml tube containing 200 μl of the magnetic beads coupled with the mAb K8/15-15. The tube was incubated for 20 min at 7°C in an automated mixer. Each tube was placed in the MPS for 2 min and while it was there, the supernatant was discarded by aspiration. The tube was removed from the MPS, and 1 ml of isolation buffer was added. The solution was pipetted 2–3 times and the tube placed in the MPS for 2 min. The supernatant was aspirated and discarded. This washing step was repeated twice. The tube was removed from the MPS, and the beads suspended with 100 μl of PBS. An aliquot of 10 μl was observed at a Nikon Eclipse-Ti microscope at 200, 400, and 600 × magnifications. The images were evaluated using phase contrast microscopy.

Cysts of *H. hammondi* and *H. heydorni* were generated in a finite bovine embryo heart cell line (KH-R; Friedrich-Loeffler-Institut, cell line No. RIE 090), as previously described (10). In brief, supernatants of the 25 cm^2^-flasks containing *H. hammondi* and *H. heydorni* cultures were individually aspirated and centrifuged at 200 *g* for 10 min. The sediment was suspended with 200 μl of isolation buffer and added to 400 μl of magnetic beads coupled to the mAb K8/15-15. The material was incubated at room temperature for 30 min in continuous agitation, and after this step, placed on the MPS. The supernatant was discarded, and the beads were re-suspended in 100 μl of isolation buffer. Two aliquots of 10 μl each were observed at the microscope. A sample of *H. heydorni* cysts was examined by immunofluorescence, but instead of a FITC anti-mouse IgG conjugate, an anti-mouse IgG coupled with a red fluorochrome was employed (Alexa fluor 555, Invitrogen).

### IMS of *in vitro* produced cysts

IMS for *in vitro* produced cysts of *T. gondii* was tested by direct and indirect methods, each one evaluated in two reaction temperatures (4 and 24°C), in total four tests. These tests were also examined using non-infected host cells (prior and post-immunomagnetic capture) to test the specificity of the method. The cells were counted in a Neubauer chamber.

### Direct capture of *in vitro* produced cysts (tests 1 and 2)

The mAb K8/15-15 was diluted 1:10 in isolation buffer (200 μl) and was incubated with 20 μl of washed magnetic beads in a tube of 1.5 ml. The tube was agitated at 4°C (test 1) or 24°C (test 2) for 40 min using an automated mixer, followed by placement of the tube on the MPS for 1 min. Then, the supernatant was collected and discarded, and the tube removed from the MPS. Isolation buffer (1.5 ml) was added to the tube, which was placed again for 1 min on the MSP. The supernatant was collected and discarded. This step of addition and removal of isolation buffer was repeated as above, and the beads were finally suspended in 1 ml of isolation buffer and stored in a sterile 1.5 ml tube at 4°C.

Pellets containing non-infected Marc-145 cells and *in vitro* produced cysts of *T. gondii* were each mixed with 250 μl of isolation buffer and 250 μl of magnetic beads coupled to mAb K8/15-15. Each suspension was placed in a 1.5 ml tube. The tubes were agitated at 4°C (test 1) or 24°C (test 2) for 20 min using an automated mixer, then, placed on MPS for 2 min. The supernatant was collected and discarded, and the magnetically attached content was saved. The tubes were removed from the MPS, and 1 ml of isolation buffer was gently added to the tube. The content was homogenized by gently pipetting the solution for three times. The tubes were placed again on the MPS for 1 min, then, the supernatant with non-attached content was collected and discarded. The tubes were submitted for an additional round of washing by adding and removing isolation buffer and using the MPS. After washing, the attached structures were homogenized with 100 μl of PBS and 30 μl from each tube were placed on three wells of teflon-coated slides for immunofluorescence evaluation.

### Indirect capture of *in vitro* produced cysts (tests 3 and 4)

Pellets containing non-infected Marc-145 cells and *in vitro* produced cysts of *T. gondii* were each vortexed with 500 μl of isolation buffer and 5 μl of mAb K8/15-15. Each tube was agitated at 4°C (test 3) or 24°C (test 4) for 10 min using an automated mixer, then, 1 ml of isolation buffer was added to each tube, followed by centrifugation (400 g, 4 or 24°C, no brake) for 10 min. After centrifugation, the supernatant was discarded, and each pellet was suspended with 200 μl of isolation buffer and 5 μl of washed beads. The tubes were agitated using an automated mixer at 4°C (test 3) or 24°C (test 4) for 20 min. Then, 1 ml of isolation buffer was added to each tube, followed by placement of the tubes on the MPS for 2 min. The tubes were removed from the MPS, and 1 ml of isolation buffer was gently added to the tube. The content was re-suspended by gently pipetting the solution for three times. The tubes were placed again on the MPS for 1 min, then, the supernatant with non-attached content was collected and discarded. The tubes were submitted for an additional round of washing by adding and removing isolation buffer and using the MPS. After washing, the remaining material was homogenized with 100 μl of PBS and 30 μl of the content from each tube were placed on three wells of teflon-coated slides for immunofluorescence evaluation.

### Separation attempt of *in vitro* produced cysts using percoll gradients

TC of *T. gondii* were generated *in vitro* as described elsewhere in this study. Host cells containing cysts of the parasite grown on a 25-cm^2^ culture flask were trypsinized, blocked by adding 2 ml of fresh medium and the washed content (1 ml) transferred to a 15-ml tube. The 1 ml solution containing cysts in host cells was homogenized with 10 ml of PBS-Tween (0.05% of Tween). Separation of cysts by Percoll gradients were conducted identically as the use of Percoll for separation of cysts produced *in vivo*. The final target fraction was resuspended with 200 μl of PBS and observed by light microscopy.

### Immunofluorescence

Teflon-coated slides containing 12 wells of 5 mm diameter each were used in immunofluorescence reactions. Wells were filled with suspensions of 10 μl of host cells containing *T. gondii* cysts before capture using antibody-coated glass beads (pre-capture) and after IMS (post-capture). The slide was dried for 15 min at 37°C and stored at −20°C until analysis. For immunofluorescence reaction, the slide was fixed in cold acetone for 5 min, immersed in PBS for 10 min and dried at room temperature. The primary antibody (mAb K8/15-15), diluted at 1:2 in PBS, was added to each well and the slide was incubated in a humid chamber at 37°C for 30 min. After incubation, the slide was immersed in a washing buffer (Na_2_CO_3_ 25 mM, NaHCO_3_ 100 mM and NaCl 35 mM, pH 9.0) for 10 min, followed by a washing in PBS for 10 min. The slide was dried at room temperature and the secondary antibody (FITC anti-mouse IgG, Sigma Aldrich, USA) was applied at 1:50 dilution and 0.05% of Evans blue. Cysts generated in cell culture of *T. gondii* and *Hammondia heydorni* were also tested as described above, but using a rabbit anti-BAG1 (16) as a primary antibody, and Alexafluor (Alexa 488) donkey anti-rabbit-IgG (1:500) as a secondary antibody. Slides were incubated in a dark and humid chamber for 30 min and washed as described for the primary antibody. Slides were dried at room temperature and mounted with glycerin (90% glycerol and 10% of PBS) and coverglass. Reactions were analyzed at a Nikon microscope and Nikon NIS-Elements software.

### Statistical analysis

Immunomagnetic capture for *in vitro* produced cysts was performed in quadruplicates, resulting in a total of 16 culture flasks for tests 1, 2, 3, and 4. The number of fluorescent cysts in three wells (total of 30 μl) was counted for each flask, and the total number of cysts per flask was extrapolated for the 100 μl solution. The cyst/ host cells ratio was determined by counting the labeled cysts and host cells in five microscopic fields (600 × magnification) of each well. Pre-capture and post-capture samples was evaluated in triplicate, resulting in a total of 96 samples. The cyst/host cells ratio was determined for the pre-capture samples. The Kruskal-Wallis test was employed to compare tests 1, 2, 3, and 4 in each pre- and post-capture samples. Differences were considered statistically significant if *p* < 0.05.

## Results

### IMS of *in vitro* produced cysts of *T. gondii*

**Figure 2:**
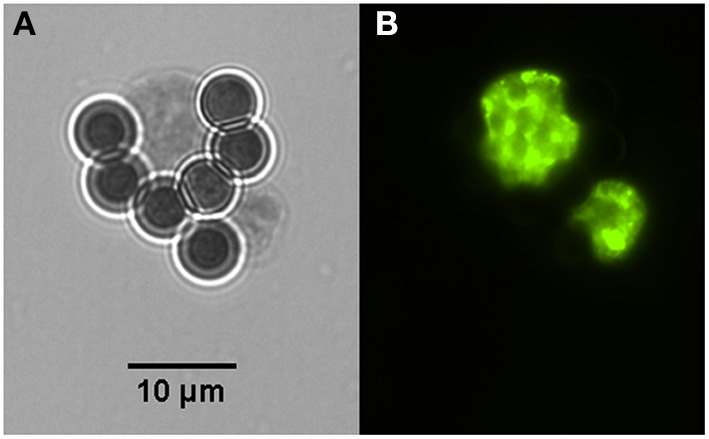
Two i*n vitro* produced tissue cysts of *Toxoplasma gondii* bound to magnetic beads containing the monoclonal antibody K8/15-15. **(A)** One of the cysts has about the size of the magnetic bead, which has 4.5 μm in diameter. **(B)** Bradyzoites inside the captured cysts were labeled by immunofluorescence using a rabbit serum to the bradyzoite antigen BAG-1.

The post-capture samples were examined by microscopic evaluation of all fields on the entire well of the slide (Table 1). The cyst/host cell ratio for pre-capture samples were obtained after evaluation by immunofluorescence of five microscopic fields using 600 × magnification. The captured cysts exhibited fluorescence in their cyst walls and magnetic beads were attached to them. Reactions were also tested using rabbit serum to BAG1 which label bradyzoites inside the cyst (Figure 2).

**Table 1:** Immunomagnetic separation of *in vitro* produced cysts of *Toxoplasma gondii* by the direct and indirect methods using two reaction temperatures.

**Number of cysts captured per flask**				
	Direct at 4°C	Direct at 24°C	Indirect at 4°C	Indirect at 24°C
	320	620	584	2,481
	330	834	396	1,590
	161	557	541	1,775
	518	570	396	2,762
Mean	332.25*	645.25	479.25	2,152*

Direct = magnetic beads anti-mouse IgG are coupled to the murine monoclonal antibody K8/15-15, and then, incubated with cyst-host cell suspension; Indirect = cyst-host cell suspension was incubated with the murine monoclonal antibody K8/15-15, and then, incubated with the magnetic beads anti-mouse IgG.

* To indicate statistically significant differences.

### IMS of *T. gondii* tissue cysts produced in mice

**Figure 3:**
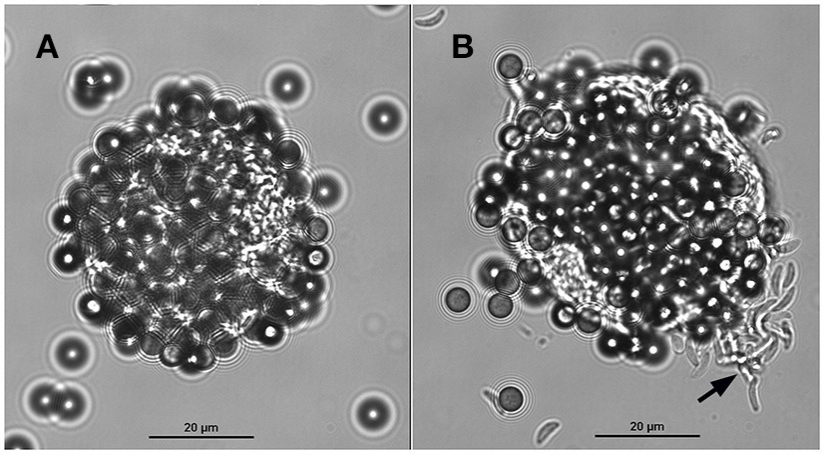
A tissue cyst of *Toxoplasma gondii* derived from mouse brain bound to magnetic beads containing the monoclonal antibody K8/15-15. **(A)** The beads cover most of the tissue cyst surface. **(B)** The same tissue cyst shown in A was mechanically ruptured by pushing the coverslip against the glass slide; note that some beads were detached from the cyst wall and numerous bradyzoites (black arrow) were released from the tissue cyst.

Tissue cysts (*n* = 1,160) were obtained from two chronically infected mice. About 50% of the tissue cysts were recovered after purification using Percoll gradients. The resultant suspension with purified tissue cysts (*n* = 500–600) contained ~9 mouse erythrocytes per tissue cyst. After incubation with the mAb-coupled beads, the final bead suspension was resuspended with 100 or 200 μl of PBS to facilitate the microscopical visualization of cyst-beads complexes. The surfaces of the tissue cysts were completely covered by the magnetic beads. To better visualize the tissue cysts which were covered by magnetic beads, and to ensure that they were indeed tissue cysts and not artifacts, the coverslip was mechanically pressed against the glass slide. The pressure on the coverslip caused the removal of part of the beads from the cyst wall and rupture the cyst, inducing the release of bradyzoites (Figure 3). No mouse erythrocytes were observed among the suspension. No further treatment was performed to separate tissue cysts from magnetic beads. For this reason, captured tissue cysts were not quantified.

### IMS of *H. hammondi* and *H. heydorni* cysts

**Figure 4:**
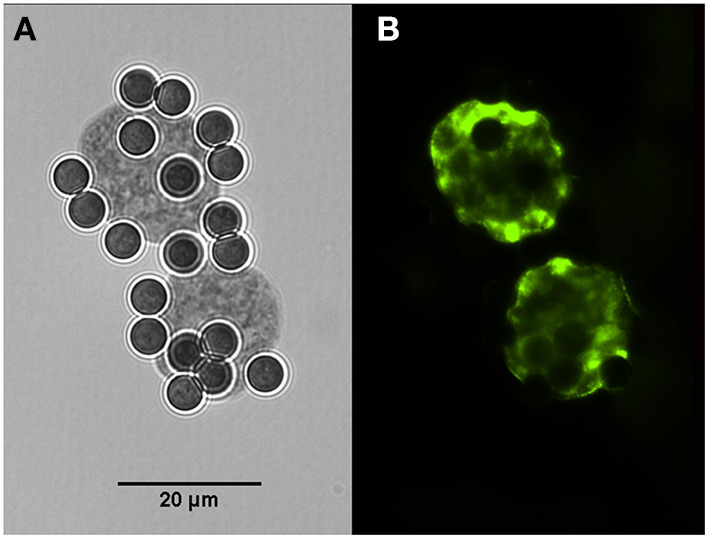
Cysts of *Hammondia heydorni* produced in cell culture and captured by magnetic beads containing the monoclonal antibody K8/15-15. **(A)** The captured cyst observed in bright field. **(B)** Immunofluorescence using a rabbit serum to the bradyzoite antigen BAG-1.

Cysts of *H. hammondi* and *H. heydorni* were obtained from supernatants of bovine heart cells (KH-R) infected with sporozoites of the parasites. After IMS, magnetic beads attached to cysts of both parasites were suspended in 100 μl solutions. Aliquots of 10 μl contained ~7–10 cysts, which varied in dimensions. Immunofluorescence was performed using captured cysts of *H. heydorni* (Figure 4).

**Figure 5:**
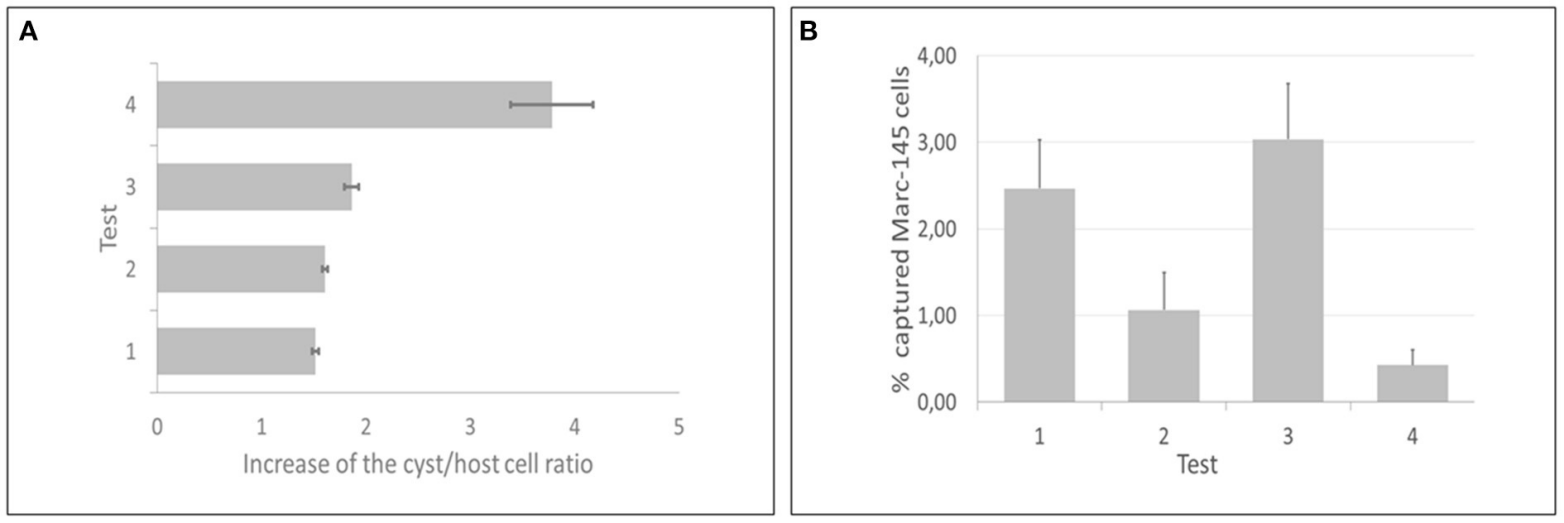
Direct and indirect immunomagnetic separation methods for *in vitro* produced cysts of *Toxoplasma gondii* were each tested at two reaction temperatures (4 and 24°C). **(A)** Test 4 (indirect capture at 24°C) presented the best performance, as the cyst/host cell ratio had a maximum increase of 3.78 times. **(B)** Test 3 (indirect capture at 4°C) showed the highest non-specific binding, contrasting with test 4, that showed the lowest non-specific binding.

All IMS methods (tests 1, 2, 3, and 4) employed in the current study showed significant increases of the cyst/host cell ratio, reaching a maximum increase of 3.78 times using test 4 (Figure 5A). Statistical difference was observed between test 4 and test 1; however, there was no statistical difference among tests 2, 3, and 4. The median of cysts isolated in each test (4 flasks per test) corresponded to 305 (test 1), 610 (test 2), 480 (test 3), and 2,170 (test 4). Non-specific capture was also examined and was based on the binding of magnetic beads to non-infected host cells (Marc-145). In all tests some degree of non-specific binding was observed. In test 3, a higher number of host cells was captured in comparison with test 4 (Figure 5B). The duration of the test for the indirect and direct method were 1 h and 1 h 10 min, respectively.

### Percoll separation of *in vitro* produced cysts of *T. gondii*

The final 200 μl suspension obtained between after Percoll separation was observed by light microscopy and contained rare cells with cyst-like structures. The cysts did not migrate to the expected fractions (30 and 90% Percoll). Microscopic examination of aliquots of the top layer, which is expected to contain cell debris, revealed host cells with cyst-like structures (parasitophorous vacuoles filled with zoites); it shows that cysts produced in cell culture presented similar densities (low density) as non-infected cells (Marc-145), and for this reason, did not migrate to 30 and 90% Percoll gradients.

## Discussion

We reported here an IMS method for isolation of tissue cysts of *T. gondii* using a murine IgG-mAb. The mAb-attached magnetic beads were demonstrated to bind to cyst walls of the parasite. To our knowledge, this is the first IMS method targeted for intact tissue cysts of *T. gondii*. The same mAb-attached beads also successfully captured cysts of *H. hammondi* and *H. heydorni* produced in cell culture.

A previous IMS method was developed for purification of *T. gondii* cyst wall using lysed tissue cysts from *in vivo* or *in vitro* produced cysts (17); the authors used magnetic beads coupled to *Dolichos biflorans* lectin (DBA), as DBA had been shown to bind to a cyst wall protein called CST1 (18). Three IMS methods have been developed, so far, to detect *T. gondii* oocysts or sporocysts in contaminated samples. In the first method, the authors conducted an indirect binding using an IgM mAb targeted to the oocyst wall of *T. gondii* (19); however, when this method was tested with turbid water containing debris, non-specific binding of debris to the coupled magnetic beads was observed. Another IMS was attempted with a different mAb-IgM directed to the sporocyst wall of *T. gondii* (20); in this method a direct binding was employed, i.e., the magnetic beads were firstly coupled to the mAb, followed by addition of the test sample. For this test, sonication of the test sample is needed, as sporocysts must be released from oocysts before adding the magnetic beads (20); sporocysts of related coccidia, such as *N. caninum* and *Hammondia* sp., are bound by the mAb, what required additional analysis of the samples by PCR (20). The third IMS consisted of an improvement of the previous methods and was based on the use of an IgM-mAb covalently bound to magnetic beads coupled to qPCR (21). This method showed promising results for detection of oocysts in contaminated fruits.

To our knowledge, no reported IMS method has been applied for intact tissue cysts of *T. gondii*. The IMS developed here, based on a murine IgG-mAb, can capture significant numbers of *T. gondii* cysts using a single culture flask of 25 cm^2^. Moreover, this IMS is also applicable to separate cysts of related coccidia, such as *H. hammondi* and *H. heydorni*, which have been used in comparative proteomic/genomic studies with *T. gondii* (22, 23). In the present work, the IMS was qualitatively tested with *T. gondii* tissue cysts produced in mice, as these cysts became available from a previous experiment (10). The major focus of this study was IMS using *T. gondii* cysts generated in cell culture. The method resulted in expressive numbers of separated cysts (>2,000) produced in cell culture and represents a promising technique for studies involving tissue cysts.

Tissue cysts of *T. gondii* have been separated for decades using Percoll gradients (24). A great recovery of tissue cysts was achieved with this method, which has been performed in different versions and applied to isolate tissue cysts from other animal species, besides mice (14, 15). The isolated cysts obtained by Percoll gradients are quite pure, with some contamination with red blood cells. An improved Percoll separation method resulted in a pure fraction of tissue cysts with no erythrocyte contamination (25). In the present work, we used Percoll gradients, which successfully allowed the separation of tissue cysts produced in mice. When we applied the same method for *in vitro* generated tissue cysts, the cysts did not migrate to the expected gradient. The density of these cysts derived from cell culture was probably lower than those produced in mice. A similar finding was reported by others (25), who observed that the use of Percoll gradients is not indicated for separation of cysts from mutant strains; these cysts are more fragile than typical ones, so they may not resist to the separation protocol or are not able to reach the expected gradient. We have not tested the density of cyst derived from cell culture, but as these cysts are produced in 4 days, they should be more fragile and present a lower density than those developed in mice.

The IMS for *T. gondii* tissue cyst in the current work was tested using direct and indirect methods, each one evaluated at two temperatures. The indirect method, which consisted of the addition of the mAb to the cell suspension, followed by inclusion of the magnetic beads to the reaction, showed the best performance. The cyst/host cell ratio was significantly increased by using the indirect method at a reaction temperature of 24°C. Although the indirect method at 24°C (test 4) did not statistically differed from the indirect method at 4°C (test 3), the latter one (test 3) showed a significant non-specific binding to Marc-145 cells. The direct method requires a longer initial incubation time (antibody plus magnetic beads) when compared with the incubation time (antibody plus target cell) of the indirect method. Therefore, the duration to perform the direct method was longer than the indirect one.

The mAb used here is of IgG class, what seems to minimize non-specific binding of the mAb to host cells or cell debris. Some additional experimental controls that were not included in our study, such as the use of magnetic beads uncoupled to mAb K8/15-15 to test non-specific binding of the beads to tissue cysts would certainly enrich the results obtained here. During the IMS for tissue cysts, caution should be taken to wash the complex bead-tissue cysts, as tissue cysts generated in cell culture are more fragile than those generated in mice. Instead of vortexing, the complexes should be washed by pipetting the sample using 1 ml-automatic pipettes.

In conclusion, we developed an IMS method based on the use of an IgG mAb targeted to tissue cyst walls of *T. gondii*. We could isolate significant numbers of cysts produced in cell culture. The method reported here should facilitate identification of molecules on the walls of *T. gondii* and related parasites. Moreover, it represents a promising alternative for using *in vivo* generated cysts, reducing the need of animal experiments.

## Data availability statement

The original contributions presented in the study are included in the article/supplementary material, further inquiries can be directed to the corresponding author.

## Ethics statement

The animal study was reviewed and approved by Landesamt für Landwirtschaft, Lebensmittelsicherheit und Fischerei of the German Federal State of Mecklenburg-Vorpommern.

## Author contributions

MR-G: conducted the experiments, analyzed the results, and wrote of the manuscript. AS: performed the statistics and revised the manuscript. JD: provided samples and revised the manuscript. GS and LG: designed the experiment, provided financial support, and revised the manuscript. All authors approved the final version of the submitted manuscript.

## Funding

MR-G was recipient of a fellowship by Coordenação de Aperfeiçoamento de Pessoal de Nível Superior (CAPES). This work was financially supported by Fundação de Amparo à Pesquisa do Estado da Bahia (FAPESB) under the Grant Number APP0053/2016. LG and AS are recipients of research productivity fellowships by Conselho de Desenvolvimento Científico e Tecnológico (CNPq). The generation of the mAb was partially funded by the German Federal Ministry of Education and Research (Toxonet01 and Toxonet02; funds to GS; 01KI0765 and 01KI1002F).

## Conflict of interest

The authors declare that the research was conducted in the absence of any commercial or financial relationships that could be construed as a potential conflict of interest.

## Publisher's note

All claims expressed in this article are solely those of the authors and do not necessarily represent those of their affiliated organizations, or those of the publisher, the editors and the reviewers. Any product that may be evaluated in this article, or claim that may be made by its manufacturer, is not guaranteed or endorsed by the publisher.
